# Social Reactions and Reasoned Pathways of High School Students and School Dropouts’ Inclination toward Smoking Behavior: Prototype/willingness Modelling via Generalized Structural Equation

**Published:** 2018-09

**Authors:** Mohammad ASGHARI JAFARABADI, Haidar NADRIAN, Hamid ALLAHVERDIPOUR

**Affiliations:** 1. Road Traffic Injury Research Center, Tabriz University of Medical Sciences, Tabriz, Iran; 2. Dept. of Statistics and Epidemiology, Faculty of Health, Tabriz University of Medical Sciences, Tabriz, Iran; 3. Dept. of Health Education and Promotion, Faculty of Health, Tabriz University of Medical Sciences, Tabriz, Iran; 4. Clinical Psychiatry Research Center, Dept. of Health Education and Promotion, Tabriz University of Medical Sciences, Tabriz, Iran

**Keywords:** School dropouts, *P*/W model, Smoke, High school students, Generalized structural equation modeling

## Abstract

**Background:**

To investigate the determinants of smoking behavior among Iranian adolescents applying the Prototype/Willingness (P/W) Model.

**Methods:**

In this cross-sectional study, a self-administered P/W model-based questionnaire was completed between 760 randomly selected adolescents (high school students and dropouts) in Hamadan, Iran, in 2015. Generalized structural equation modeling (GSEM) was applied to analyze data.

**Results:**

Significant associations were found between subjective norms and positive attitudes toward smoking (*P*<0.001). The behavioral intention was also significantly related to the willingness and subjective norms (*P*<0.001). Prototype or risk image was not significantly related to the willingness among the dropout adolescents.

**Conclusion:**

When social reaction and reasoned processes are modeled together, both may predict the smoking behavior. The high-risk perception and the high-risk image toward smoking behavior among the adolescents may originate from socio-cultural factors underlying the behavior. Further research is recommended to investigate the socio-cultural biases of the issue.

## Introduction

If the current worldwide patterns of smoking continue, one billion deaths are expected to happen in this century from tobacco use, the most of which is low- and middle-income countries (1–3). Smoking is primarily initiated during adolescence (4). Studies have reported different prevalence rates for smoking among adolescents, ranging from 9.3% to 39% (4–7). The prevalence rate of smoking behaviour among Iranian adolescents (9.5% to 26.0%) is alarming (8–12).

Theory-based approaches (13) like the theory of reasoned action (TRA) and the Theory of planned behavior (TPB) explain risky behavior from a decision-making perspective and assume that behavior is intentional (14). The applicability of these approaches is controversial (15, 16) and evidence has demonstrated the application of theories emphasizing social interaction and reasoned path perspectives in explaining the willingness and intention to smoke, respectively (13). The decision of an adolescent for smoking is influenced by his/her smoking images (16, 17).

### Prototype/Willingness Model

**Fig. 1:**
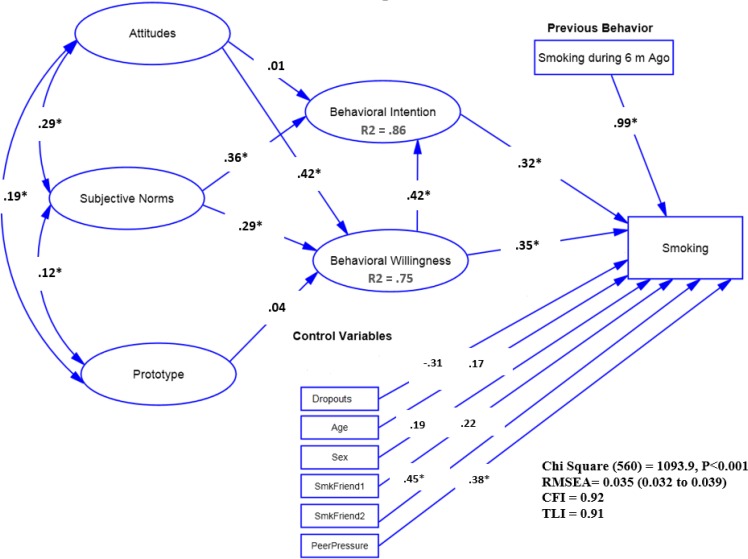
The relationship among study variables reflected in P/W full model *: *P*<0.05: the model parameters were also adjusted for control variables. Employee Status (1: Employed (Dropouts), 2: Un-Employed (Dropouts), Reference Category: Students). Age: Age Category (Comparing: 17 and higher age with Reference Category of 16 and lower). Sex (Comparing Boys with Reference Category of Girls). SmkFriend: Having Smoker Friend (1: Always, 2: Sometimes, Reference Category: Not Have). PeerPressure: Peer Pressure (Comparing Yes with Reference Category of No)

The Prototype/Willingness (P/W) model was developed to explain and predict risky behaviors among adolescents (Fig. 1) (18), assuming two pathways: 1) the reasoned action path reflects a conscious decision-making process engaging a risky behaviours and 2) the social reaction pathway, which is much more reactive and unintentional (19) and is a response to the situations that are conducive toward risk (20).

One of the central constructs in the P/W model is behavioral willingness (BW) which explains the unintentional or reactive component of a risk behavior and is defined as openness to risk opportunity (18).

Willingness to engage in a risky behavior usually develops before intentions (21). Both BW and behavioral intentions (BI) may predict risk behaviors (18, 22).

The second major key construct in the model is prototype image refers to one’s perception from typical individuals of his/her age who engage in a behavior. The prototype is positively related to willingness for engaging behavior (22–24).

In the current study, we examined the application of P/W model to explain smoking among Iranian adolescents. Several studies in the developed countries have suggested different pathways for smoking initiation (25–29) which may be different from those in developed countries due to the differences may exist in the cultural values, socioeconomic status, and racial/ethnic groups. In Iran, several studies have assessed the determinants of smoking among adolescents (10, 30). To the best of our knowledge, no study has focused on the issue, therefore, understanding the cognitive determinants of adolescents’ decisions to engage in tobacco smoking applying the PW model may be helpful aiming at smoking cessation among adolescents.

The objective of this study was to investigate the determinants of smoking behavior among Iranian adolescents applying the P/W model considering the possible differences in social reaction pathway among adolescents by employment status and gender utilizing GSEM.

## Materials and Methods

### Participants

This cross-sectional study was conducted on 760 high school students (including the dropouts from the schools) with 14 to 18 yr of age in Hamadan, Iran, in 2015. Cluster random sampling was employed to recruit the 9^th^ to 12^th^-grade high school male and female students. Eight high schools were randomly selected from the educational districts of Hamadan. Twenty male and 15 female students were randomly selected from each of the four educational grades to complete the questionnaire. The employed dropouts were randomly chosen from their location of employment and the unemployed students were recruited from the streets and public parks.

This study was conducted after providing approval from the Institutional Review Board of Hamadan University of Medical Sciences. Informed assent and consent were obtained from all the participants. The students were assured of the complete anonymity of their data.

### Instruments

***Willingness to smoke cigarettes***: The questionnaire was started with presenting a hypothetical risk-conducive situation as follows: “Suppose you were with a group of friends some of whom were smoking and there were some extra cigarettes for you to smoke if you wanted.” Then, three different questions regarding willingness to smoke were presented on how they would react in such a situation (31). The response to each item was recorded on a 5-point Likert-type scaling from 1 (not at all) to 5 (extremely willing” respectively (the total score range = 3–15). The higher score indicated the higher level of willingness to smoke.

***The prototype of smokers:*** The participants were first asked to imagine a classmate or friend in their age who smokes cigarettes. Then participants were asked to rate the favorability of the image by 7 adjectives (popular, smart, good-looking, cool, childish, careless, and dull/boring) (31). A reverse scale was employed for the latter 4 adjectives. Response format was based on a 5-point scale ranging from 1 (not favorable at all) to 5(extremely favorable). Higher values indicated a positive image of smoking.

***Intention to smoke cigarettes:*** The behavioral intention to smoke was a self-administered scale including 5 items. An example of the items is as follows: “I intend to smoke in the next month.” The response format was based on a 5-point scale ranging from 1 (not at all) to 5 (absolutely).

***Subjective Norms about cigarette smoking:*** A 3-item scale was developed to evaluate subjective norms. An example of the items was “I think that I should leave my friends who smoke?” The response format was based on a 5-point scale ranging from 1 (completely disagree) to 5 (completely agree).

***Positive attitude toward cigarette smoking:*** Positive attitude toward smoking was 10 items developed to assess the personal beliefs on smoking. One of the items, for instance, was “Smoking will help me to be relaxed.” The response format was based on a 5-point scale ranging from 1 (totally agree) to 5 (totally disagree). Two items were reversely designed and needed to be recorded before data analysis. The higher scores indicated a more positive attitude toward cigarette smoking.

***Smoking Behaviour:*** This measure included one item: “Have you ever smoked cigarettes?” A dichotomous answer (yes/no) was considered as the response format.

***Demographics and Tobacco Smoking Related Variables:*** The background variables included age, gender, employment status (students\employed\unemployed), the history of tobacco smoking in the prior month (yes/no), and prior 6 months (yes/no), age at time of smoking initiation (year), parents smoking (yes/no), friends smoking (yes/no), peer persuasion and pressure to smoke (yes/no).

***Validity and reliability of the Measures:*** The measure showed a good to excellent ranges of internal consistency for the scales: willingness (α = 0.92), prototype (α= 0.79), intention (α= 0.92), subjective norms (α= 0.78), and Attitude (α= 0.86). Construct validity of the measure was assessed and confirmed applying confirmatory factor analysis (CFA).

### Statistical Analyses

Statistical analyses were performed using M*Plus* (6.2) (32) and SPSS (17) (SPSS Inc., Chicago, IL, USA). To test the construct validity and the fitness of the measurement model and to fit the conceptual P/W model to data, CFA and GSEM were used, respectively (32). To investigate the fitness, the goodness of fit indices was calculated. Values smaller than 0.08 for root mean square error of approximation (RMSEA) and values greater than 0.90 for Tucker-Lewis index (TLI) and comparative fit index (CFI) confirmed the fitness of model (33). Additionally, multi-group GSEMs were conducted by educational status and gender.

## Results

### Study participant characteristics:

Among all the 760 adolescents aged 14–18 yr, 71% were enrolled in an academic institution and the remaining (29%) were dropouts ((employed (21%) and unemployed (8%)). More than 39% were 16 yr old or younger. Moreover, 51% were male and 49% were female. Almost 27% of the participants were smokers and the mean age of smoking initiation was 13.9 ± 2.2 yr (Table 1).

**Table 1:** Demographic and background characteristics of the participants

***Variables***	***Frequency***	***Percent***
**Employee Status**		
Student	537	70.7
Employed	161	21.2
Unemployed	62	8.2
**Age**		
<= 16 (yr)	297	39.1
>17 (yr)	463	60.9
**Gender**		
Male	388	51.1
Female	372	48.9
**Tobacco Smoking**		
No	549	74.4
Yes	189	25.6
**Peer Pressure**		
No	574	85.2
Yes	100	14.8
**Smoking in the prior six months**		
No	498	79.3
Yes	130	20.7

***Full Model:*** The full model fitted the data well after some modifications; χ^2^_(326)_ =1093.9, *P*<.001, χ^2^/df =1.9<5, TLI= .91>0.9, CFI= .92 >0.9, RMSEA=0.035< .08 (90% CI =(0.032 to 0.039). In addition, all the relationships between the items and the scales were statistically significant (*P*<0.001). Findings showed no significant relationship between the adolescents’ prototype and their willingness (*P*=0.193). However, the subjective norms (*P*<0.001) and the positive attitudes (*P*<0.001) had significant and positive relationships with the willingness. The intention was also significantly related to willingness (*P*<0.001) and subjective norms (*P*<0.001). Finally, in this model, the relationships between smoking with willingness (*P*=0.005) and behavioral intention (*P*=0.016) were positively significant (Fig. 1).

**Fig. 2:**
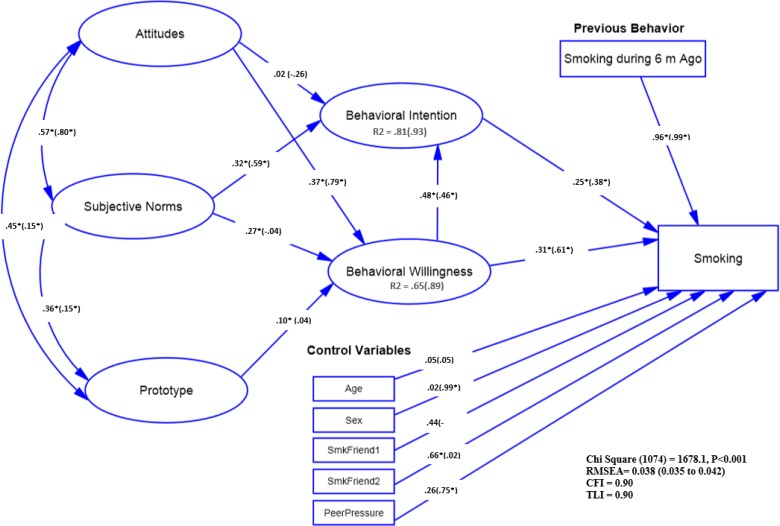
The relationship among study variables reflected in P/W full model by employee status (Students v. s. Dropouts (Employed and Un-Employed). *: *P*<0.05 The model (adjusted for control variables) parameters were presented for students outside of parentheses and for employed and unemployed subjects inside of the parentheses. Age: Age Category (Comparing 17 and higher age with Reference Category of 16 and lower). Sex (Comparing Boys with Reference Category of Girls).SmkFriend: Having Smoker Friend (1: Always, 2: Sometimes, Reference Category: Not Have). PeerPressure: Peer Pressure (Comparing Yes with Reference Category of No)

***Multi-group model by educational status:*** The model fit well and showed a significant difference between students and dropouts (*P*<0.001). Among students, there was a significant inverse relationship between the smoker prototype (*P*=0.017) and subjective norms (*P*<0.001) with willingness. In addition, the attitudes were significantly related to willingness among students and dropouts (*P*<0.001). Furthermore, a significant relationship was found between intention with willingness and subjective norms among students and dropouts (*P*<0.001). Finally, positive and significant relationships were found between smoking with willingness and intention among students and dropouts (*P*<0.05) (Fig. 2).

***Multi-group model by gender:*** The model fit well and gender and showed a significant difference between girls and boys (*P*<0.001). Smoker prototype and willingness were not significantly related among both genders (*P*>0.05). Significant positive relationships were found between subjective norms and willingness among boys and girls (*P*<0.05).

In both groups, the attitude and intention were significantly related to willingness (*P*<0.001).

**Fig. 3:**
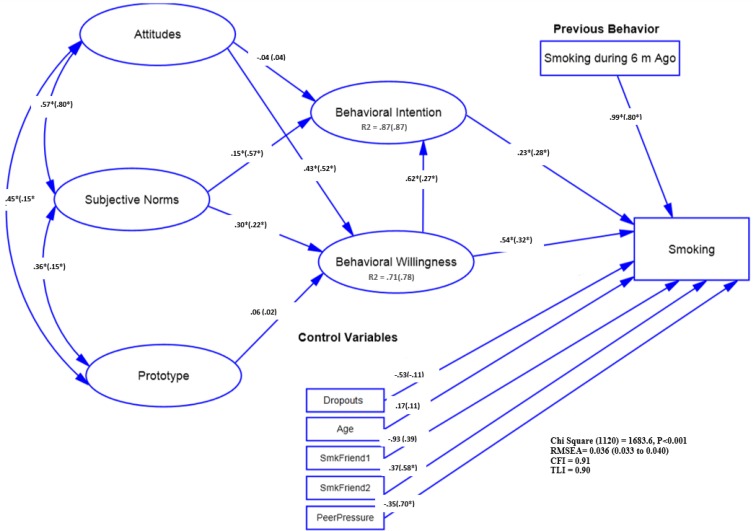
The relationship among study variables reflected in P/W full model by Sex *: *P*<0.05 The model (adjusted for control variables) parameters were presented for Girls out of parentheses and for Boys in the parentheses. Employee Status (1: Dropouts (Employed and Un-Employed), Reference Category: Students). Age: Age Category (Comparing: 17 and higher age with Reference Category of 16 and lower). SmkFriend: Having Smoker Friend (1: Always, 2: Sometimes, Reference Category: Not Have). Peer Pressure: Peer Pressure (Comparing Yes with Reference Category of No)

Such associations were also found between intention and subjective norms (*P*<0.001). The smoking and willingness were also positively associated among boys and girls (*P*<0.05) (Fig. 3).

## Discussion

This is the first study that explored the applicability of the P/W model for predicting smoking behavior among adolescents. The social reaction path was mediated by attitudes towards cigarette smoking. Moreover, the social norms on tobacco smoking affected the behavioral willingness and consequently smoking behavior which was consistent with those found in a previous study (34). The relationship between social norms and smoking was mediated by intention. Both intention and willingness demonstrated significant relationships with smoking. The P/W model could be a useful theoretical framework to describe how the willingness and behavioral intention may predict and mediate smoking behavior among adolescents.

***Full P/W model:*** We found willingness and behavioral intention as two main predictors of cigarette smoking among adolescents. There is plenty of evidence suggests that willingness to engage in a risky behavior usually develops before intentions and maybe a better predictor of such behaviors among adolescents (14, 22, 23).

Several studies have reported positive attitude as a predictor of intention (35, 36). However, the extent to which the attitude positivity promotes or negativity deters smoking remains unclear (37). Attitude was related to intention to smoke (30). In our study attitude did not predict intention directly. The indirect pathway between attitude and intention found mediated via subjective norms and willingness.

The direct association of subjective norms, as a predictor for willingness, indicated that subjective norms acted as one of the main factors to predict smoking mediated via willingness. Subjective norms are associated with both greater intention and willingness (23). Smoking complies with a social nature among Iranian adolescents which makes them more susceptible to social effects rather than individual’s personal attitudes.

TRA predicted intention more than willingness (38). Dissimilarly, our findings showed that the TRA constructs predicted the willingness but not the intention. Based on TRA, attitude and subjective norms are considered as the proximal determinants of behavioral intention. However, in our findings, attitude was not proximally related to intention, and instead, subjective norms and willingness were the main predictors of intention. This finding indicated the applicability of subjective norms and willingness in promoting the intention of adolescents do not smoke cigarette.

Surprisingly, the prototype was not significantly associated with the willingness, however, was indirectly associated with willingness through attitude and subjective norms. Favorable prototypes may be associated with high willingness to engage in different behaviors (35, 39). Our inconsistent findings may be due to less broadcasting of effective risk message about smoking and also developing unelaborated risk messages especially for teenagers in the Iranian society.

We found significant relationships between intention and willingness. Social reaction path alone as a possible way of smoking behavior among adolescents (20, 39). However, our results suggest that when social reaction and reasoned processes are modeled together, both may predict the smoking behavior. It is not well known that to what extent the social reaction or reasoned processes may be attributed to initiation of smoking among Iranian adolescents. Further studies to investigate such attributions are suggested.

***Employee Status:*** Among dropouts, the social reaction processes was a stronger predictor of smoking, however, the risk images of the students were more favorable than dropouts. Additionally, among students, the smokers’ prototype was associated more strongly with attitude and subjective norms. As we expected, attitude and subjective norms were associated with willingness among students. However, among dropouts attitude was the only factor associated with willingness.

***Gender:*** Being female dominates the social reaction processes for smoking. However, among both girls and boys, both intention and willingness were significant predictors of intention to smoke. In the line with other studies (15, 20), when gender-specific images were assessed, no significant association was found between prototype and willingness. Due to the specific values and beliefs existing in the Islamic countries, the communication of a gender with the opposite gender is socially stigmatized, which may thus affect smoking behavior. In the present study, the P/W model hypotheses were supported among girls, except for one: the prototype predicted better attitude toward smoking and subjective norms.

## Limitations and Strengths

Firstly, the cross-sectional design of this study limits the ability to conclude causal inferences among model relation. As a second limitation and similar to many studies (10, 40, 41), we assessed smoking by asking one question. Thirdly, we relied on the self-report evaluation; however assuring anonymity may have lessened the inaccurate reporting (20). Fourth, the study was performed in a city in Iran which may limit the generalizability of the results.

## Conclusion

The P/W model was helpful in identifying the social reactions and reasoned pathways of smoking. When social reaction and reasoned processes are modeled together, both may predict the smoking behavior. The high-risk perception and image toward smoking among adolescents may originate from socio-cultural factors underlying the behavior. Further research is recommended to investigate the socio-cultural biases of the issue. Healthcare providers and health policymakers should consider the dominance of social reaction processes among females while designing adolescent smoking cessation programs. The social reaction processes should be also considered as a stronger determinant of smoking behavior among dropouts compared to students.

## Ethical considerations

Ethical issues (Including plagiarism, informed consent, misconduct, data fabrication and/or falsification, double publication and/or submission, redundancy, etc.) have been completely observed by the authors.
